# Mitochondrial DNA Variants Mediate Energy Production and Expression Levels for CFH, C3 and EFEMP1 Genes: Implications for Age-Related Macular Degeneration

**DOI:** 10.1371/journal.pone.0054339

**Published:** 2013-01-24

**Authors:** M. Cristina Kenney, Marilyn Chwa, Shari R. Atilano, Janelle M. Pavlis, Payam Falatoonzadeh, Claudio Ramirez, Deepika Malik, Tiffany Hsu, Grace Woo, Kyaw Soe, Anthony B. Nesburn, David S. Boyer, Baruch D. Kuppermann, S. Michal Jazwinski, Michael V. Miceli, Douglas C. Wallace, Nitin Udar

**Affiliations:** 1 Gavin Herbert Eye Institute, University of California Irvine, Irvine, California, United States of America; 2 Cedars-Sinai Medical Center, Los Angeles, California, United States of America; 3 Retina-Vitreous Associates Medical Group, Beverly Hills, California, United States of America; 4 Tulane Center for Aging and Department of Medicine, Tulane University, New Orleans, Louisiana, United States of America; 5 Children's Hospital of Pittsburgh, Pittsburgh, Pennsylvania, United States of America; Louisiana State University Health Sciences Center, United States of America

## Abstract

**Background:**

Mitochondrial dysfunction is associated with the development and progression of age-related macular degeneration (AMD). Recent studies using populations from the United States and Australia have demonstrated that AMD is associated with mitochondrial (mt) DNA haplogroups (as defined by combinations of mtDNA polymorphisms) that represent Northern European Caucasians. The aim of this study was to use the cytoplasmic hybrid (cybrid) model to investigate the molecular and biological functional consequences that occur when comparing the mtDNA H haplogroup (protective for AMD) versus J haplogroup (high risk for AMD).

**Methodology/Principal Findings:**

Cybrids were created by introducing mitochondria from individuals with either H or J haplogroups into a human retinal epithelial cell line (ARPE-19) that was devoid of mitochondrial DNA (Rho0). In cybrid lines, all of the cells carry the same nuclear genes but vary in mtDNA content. The J cybrids had significantly lower levels of ATP and reactive oxygen/nitrogen species production, but increased lactate levels and rates of growth. Q-PCR analyses showed J cybrids had decreased expressions for CFH, C3, and EFEMP1 genes, high risk genes for AMD, and higher expression for MYO7A, a gene associated with retinal degeneration in Usher type IB syndrome. The H and J cybrids also have comparatively altered expression of nuclear genes involved in pathways for cell signaling, inflammation, and metabolism.

**Conclusion/Significance:**

Our findings demonstrate that mtDNA haplogroup variants mediate not only energy production and cell growth, but also cell signaling for major molecular pathways. These data support the hypothesis that mtDNA variants play important roles in numerous cellular functions and disease processes, including AMD.

## Introduction

Mitochondria provide critical cellular energy using the tricarboxylic acid (TCA) cycle, oxidative phosphorylation (OXPHOS), and beta-oxidation of fatty acids for metabolism, cell division, production of reactive oxygen species (ROS), and apoptosis. Human mitochondrial (mt) DNA forms a circle of double stranded DNA with 16,569 nucleotide pairs. The non-coding mtDNA Dloop contains 1121 nucleotides and is important for replication and transcription. The coding region of mtDNA encodes for 37 genes including 13 protein subunits essential for OXPHOS, 2 ribosomal RNAs, and 22 transfer RNAs [1]–[3]. The mtDNA can be categorized into haplogroups that are defined by a set of specific SNP variants that have accumulated over tens of thousands of years and correspond to different geographic populations of the world. The H haplogroup is the most common European haplogroup, while the J haplogroup originates from the Northern European region and is defined by SNP variants that are associated with heat production as an adaptation to colder climates [4].

The mtDNA plays an important role in aging and diseases [4]–[6]. Specific haplogroups are associated with a variety of eye diseases including age-related macular degeneration (AMD) [7]–[10], diabetic retinopathy [11], pseudoexfoliation glaucoma [12], [13], primary open-angle glaucoma [14], keratoconus [15], multiple sclerosis-related optic neuritis [16], [17], and Leber hereditary optic neuropathy [1], [18]. In AMD, the J, T and U haplogroups are high risk while the H haplogroup is protective against developing the disease [7]–[10], [19]. The actual cellular mechanisms or pathways by which mtDNA variants contribute to disease states have not been identified. One difficulty in studying the functional consequences of different mtDNA SNP variants is that the vast majority of proteins which contribute to energy biogenesis are encoded by nuclear DNA and imported into the mitochondria [4], [20]. The contribution to energy production by the mtDNA is relatively small by comparison and experimental models have not been available to identify the nuclear-mitochondria interactions. In the present study, we have developed a human ARPE-19 cell cybrid (cytoplasmic hybrid) model, a system where individual cell lines have mitochondria representing H or J haplogroups from different individuals but contain identical nuclei. Using this model, we have taken a systematic approach to understand some of the basic mechanisms related to cellular functions that are potentially different within the H and J haplogroups. Our results show that the H and J haplogroups have different rates of energy production, cell growth, ROS production, and altered expression of nuclear genes involved in inflammation and human retinal diseases. Our findings are significant because they demonstrate that mtDNA mediates not only energy production but also cell signaling for major molecular pathways. These data support a paradigm shift in thinking about the role that mitochondria play in numerous cellular functions and disease processes.

## Materials and Methods

### Ethics Statement

All research involving human participants was approved by the Institutional review board of the University of California, Irvine (#2003-3131). Written informed consent was obtained and all clinical investigations were conducted according to the principles expressed in the Declaration of Helsinki.

### Cybrid Cultures and Culture Conditions

For DNA analyses, 10 ml of peripheral blood was collected via venipuncture in tubes containing 10 mM EDTA. DNA was isolated with a DNA extraction kit (PUREGENE, Qiagen, Valencia, CA). Platelets were collected in tubes containing 3.2% sodium citrate, isolated by a series of centrifugation steps, and final pellets were suspended in Tris buffered saline. The ARPE-19 cells deficient in mtDNA (Rho0) were created by serial passages in low dose ethidium bromide [21]. Cybrids were produced by polyethylene glycol fusion of platelets with Rho0 ARPE-19 cells according to modified procedures of Chomyn [22]. Verification of transfer of the mitochondria into the Rho0 ARPE-19 cells was accomplished by using polymerase chain reaction (PCR), restriction enzyme digestion, and sequencing of the mtDNA to identify the mitochondrial haplogroup of each cybrid [9]. The SNPs defining the J haplogroup were G13708A, C16069T and T16126C. The H defining SNPs were T7028C and A73G. All experiments used passage 5 cybrid cells for the assays described below.

### Identification of Cybrid Haplogroups

Cybrid DNA was extracted from cell pellets using a spin column kit (DNeasy Blood and Tissue Kit, Qiagen) and quantified using the Nanodrop 1000 (Thermo Scientific, Wilmington, DE). Restriction enzyme digests were performed as described in Udar and coworkers to determine mitochondrial haplogroups [9]. The digestion products were run on 3% agarose gels for the H cybrids and 1.2% agarose gels for the J cybrids. Allelic discrimination was also performed to confirm the haplogroups. The primers for allelic discrimination were synthesized by ABI Assay-by-Design. The samples were run at GenoSeq, the UCLA Genoytyping and Sequencing Core, on an ABI 7900HT. The data was analyzed with Sequence Detection Systems software from ABI.

### Reactive Oxygen/Nitrogen Species (ROS/RNS) Assay

The H haplogroup and J haplogroup cybrid cultures were incubated in 24 well plates (1×10^5^ cells/well). After 24 hours, the cells were exposed to the reagent and ROS/RNS production measured with a scanning unit (FMBio III, Hitachi, San Francisco, CA) for the fluorescent dye 2′,7′-dichlorodihydrofluorescein diacetate (H_2_DCFDA, Invitrogen-Molecular Probes, Carlsbad, CA) using 490 nm for the emission and 520 nm for the excitation wavelengths. The values represent combined results of experiments using three different H cybrids and three different J cybrids, each experiment repeated twice and the assays run in quadruplicate.

### ATP Production Assay

Intracellular ATP levels were measured for H and J cybrids using the luminescence ATP detection assay (ATPlite Perkin Elmer Inc., Waltham, MA USA) as per the supplier's instructions. Cybrid lines (H cybrids n = 3, J cybrids n = 3) were cultured 24 hours on a 96 well plate at 3 different concentrations, 100K, 50K, and 10K cells per well with a final volume of 100 µl/well. Luminescence was measured using a Synergy HT Multi-Mode microplate reader and Gen5 Data Analysis software (BioTek instruments, Winooski, VT USA). All experiments were repeated twice and assayed in quadruplicate.

### Lactate Assay

Lactate concentrations in the samples were measured by the Lactate Assay Kit (Eton Bioscience Inc, San Diego, CA). Cells were plated at 100K and 50K in 96-well plates and incubated overnight. Lactate levels were measured according to the manufacturer's protocol. Standards and samples were set up as duplicates and quadruplicates and experiments were repeated twice.

### Growth Curve Assay

The growth curves of six different cybrids were assessed over six days under similar environmental conditions. Three different H cybrids and three J cybrids were grown to passage 5 using methods described above. For each time point, 300K cells per well were plated onto six-well plates and each cybrid cell line was assayed in duplicate. Cells were incubated in standard conditions and culture medium was changed every other day. The cell numbers were measured using a Cell Viability Analyzer (ViCell, Beckman Coulter, Miami, FL). The numbers of cells plated at timepoint 0 were designated as 100% and the percentage increase in growth for each cybrid at 2, 4, and 6 days were calculated. A mean percentage increase value of all three H cybrids and three J cybrids were compared by nonlinear regression analysis (Prism, version. 3.0; GraphPad Software Inc., San Diego, CA). Within each experiment, the assays were run in duplicate and the experiments repeated twice.

### Isolation of RNA and Amplification of cDNA

Cells from cybrid cultures (H cybrids, n = 3, J cybrids, n = 3) were pelleted, and RNA isolated using the RNeasy Mini-Extraction kit (Qiagen, Inc.) following the manufacturer's protocol. The RNA was quantified using a NanoDrop 1000 (ThermoScientific). For Q-PCR analyses, 100 ng of individual RNA samples were reverse transcribed into cDNA using the QuantiTect Reverse Transcription Kit (Qiagen).

### Gene Expression Assay and Statistical Analyses

RNA was isolated as described above. For the gene expression analyses, the RNAs from the three H haplogroup cybrid cultures were combined (250 ng/µl per sample) into a single sample for analyses. Then three J haplogroup cybrid cultures were also combined into one sample. The H cybrid and J cybrid RNAs were sent to the UCLA Clinical MicroArray Core Lab for analyses with the Affymetrix Human U133 Plus 2.0 Array. The gene expression results were analyzed with pathway analysis software (INGENUITY Systems, Redwood City, CA).

### Quantitative PCR (Q-PCR) Analyses

Q-PCR was performed using primers for genes associated with AMD and retinal disorders (CFH, C3, EFEMP1, ARMS2, MYO7A, BBS10), antioxidant enzymes (SOD1, SOD2, SOD3, PRDX6, GPX3), and genes related to metabolic homeostasis (FOXO1, ME3, GFM1) (QuantiTect Primer Assay, Qiagen). Total RNA was isolated from pellets of cultured cells of haplogroup J cybrids, n = 3 and haplogroup H cybrids, n = 3 as described above. The Q-PCR was performed using a QuantiFast SYBR Green PCR Kit (Qiagen) on a Bio-Rad iCycler iQ5 detection system and expression levels were standardized for all primers using TATA box binding protein (TBP) as the reference gene. The analyses were performed in triplicate. Statistical analyses of gene expression levels were performed to measure difference between haplogroups using Prism version 5.0 (GraphPad Software, Inc., San Diego, CA).

### Statistical Analyses

Data were subjected to statistical analysis by ANOVA (Prism, version. 3.0; GraphPad Software Inc.). Newman-Keuls multiple-comparison test was done to compare the data within each experiment. P<0.05 was considered statistically significant. The p values are designated as the following: *p<0.05; **p<0.01; ***<0.001. Error bars in the graphs represent SEM (standard error mean). Experiments were performed two or three times and each treatment run in quadruplicate.

## Results

**Figure 1:**
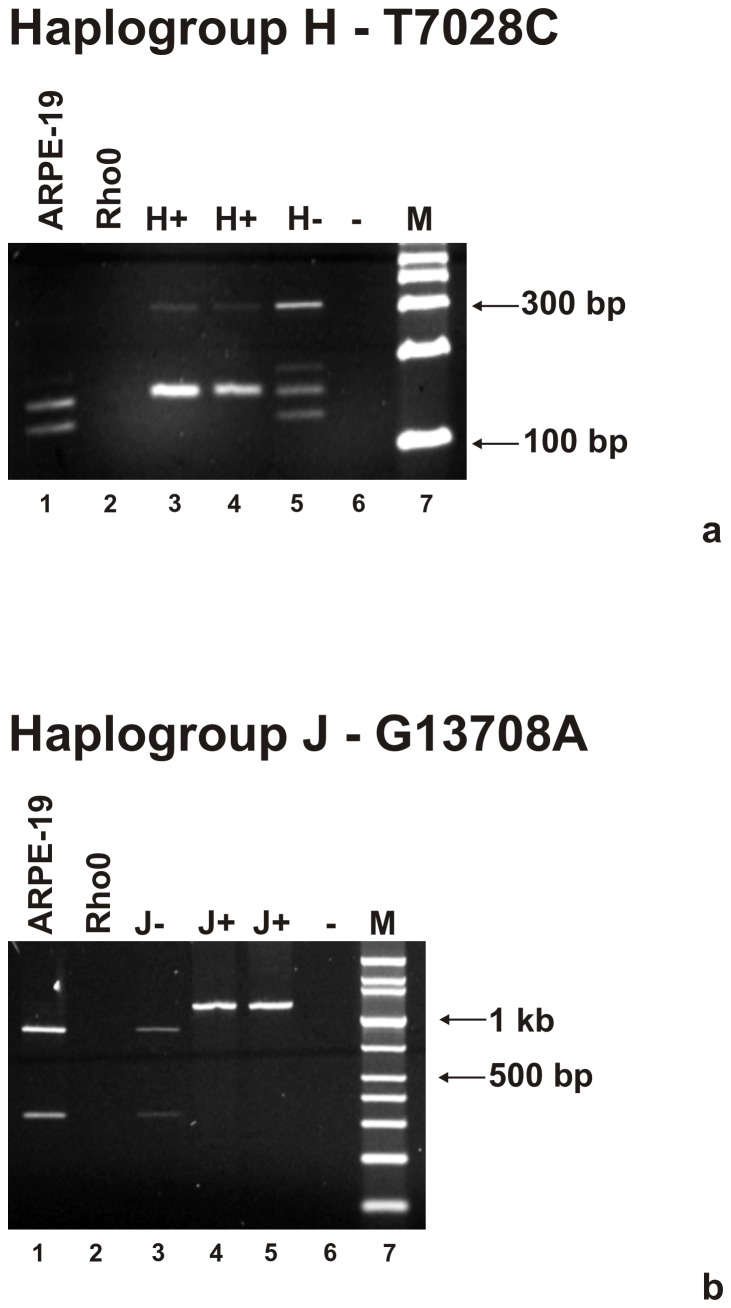
Agarose gels showing bands obtained after PCR amplification and restriction enzyme digestion of the H haplogroup and J haplogroup cybrids. Figure 1a: Panel Upper – Digestion pattern of T7028C that defines the H haplogroup appears in lanes 3 and 4. The non-H haplogroup samples appear in lanes 1 and 5. Figure 1b: Panel Lower – Digestion pattern of G13708A that defines the J haplogroup is found in lanes 4 and 5. The non-J samples are in lanes 1 and 3. The Rho0 sample lacks mtDNA PCR amplification product (lane 2, upper and lower panels). M = marker; - indicates water only; bp, basepair.

Studies were designed to first confirm the identity of each of the cybrids created from the common Rho0 cell line. Figure 1a shows that after PCR amplification and digestion with AluI enzyme, the H cybrids have mtDNA with the C allele in the SNP 7028 representing the H haplogroup (156 bp+152 bp bands; lanes 3 and 4). The non-H haplogroup mtDNA, represented by the T allele, shows bands at 152 bp+126 bp (lanes 1 and 5). Figure 1b shows the restriction digest with BstNI enzyme and reveals a single band at 1210 bp representing the A allele of J haplogroup mtDNA (lanes 4 and 5). The non-J haplogroup is represented by the G allele (874 bp+336 bp, lanes 1 and 3). The mtDNA is absent in the Rho0 cells (lane 2). Each donor mitochondrial haplogroup matched up with the corresponding cybrid, confirming their identity.

**Figure 2:**
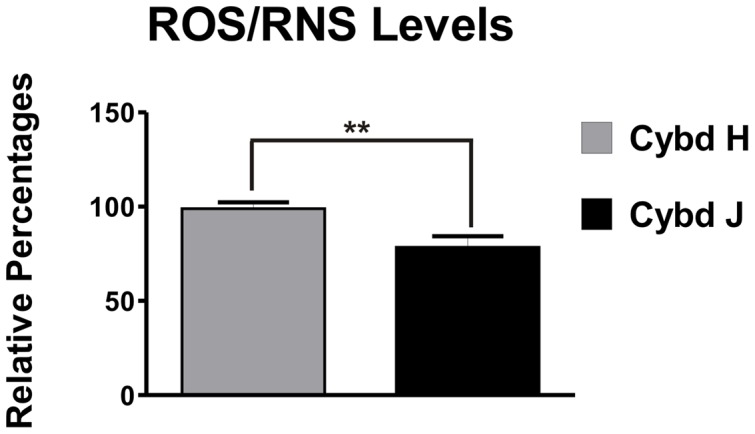
Differences in relative ROS/RNS levels are shown in H cybrid versus J cybrid cultures after 24 hours. Cybrids H produced increased levels of ROS/RNS compared to the J cybrids (**p<0.01). ROS/RNS, reactive oxygen/nitrogen species; Cybd, cybrid.

**Figure 3:**
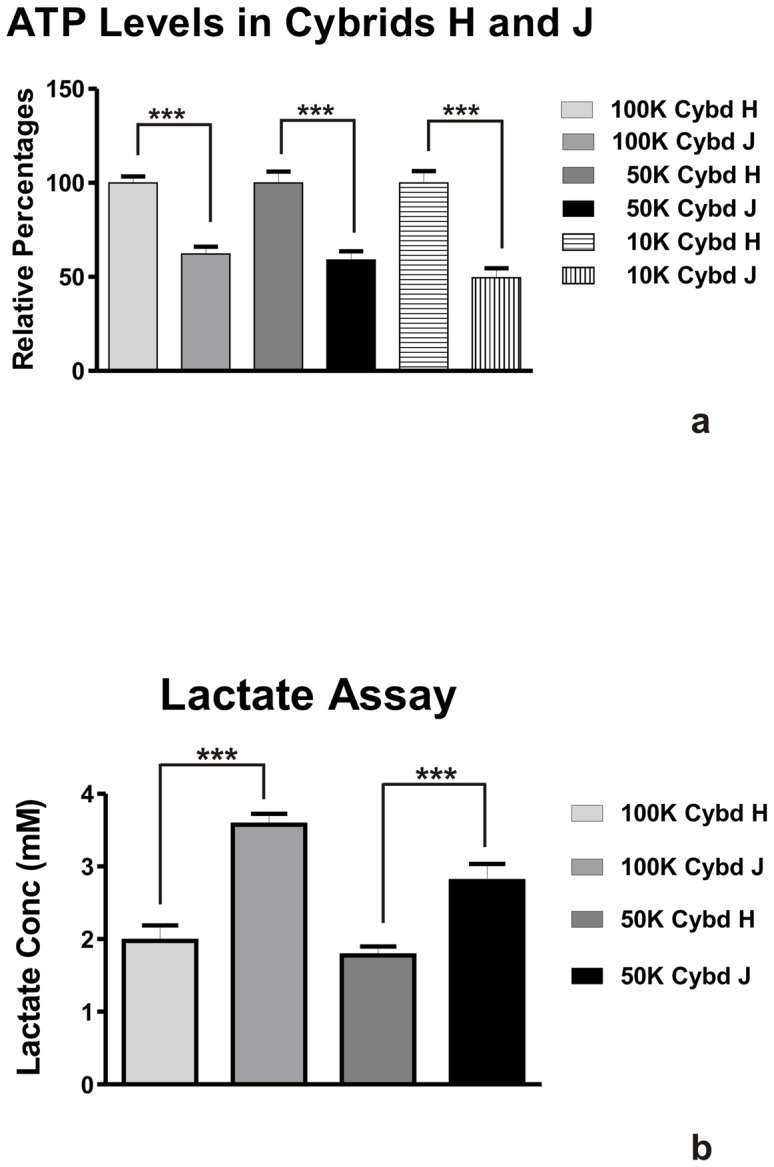
The H cybrid versus J cybrid cultures show differences in the ATP production and lactate levels. **Figure 3a** **.** Relative ATP levels in H versus J cybrid cultures at three different cell concentrations incubated for 24 hours. The H cybrids produced increased ATP levels compared to J cybrid cultures (***p<0.001). Figure 3b. Lactate levels in cybrid H versus cybrid J cultures at two different cell concentrations incubated for 24 hours. The H cybrids had decreased lactate production when compared to the J cybrids (**p<0.01, ***p<0.001) indicating lower glycolysis activity. Cybd, cybrid.

Biochemical parameters related to mitochondrial function (production of ROS/RNS, ATP, and lactate) were measured for the different cybrids. It was shown that the H haplogroup cybrids produced 21% more ROS/RNS than the J haplogroups cybrids (100±3.4% versus 79±6.1%, p = 0.006, Figure 2). Next, the cybrids were plated in concentrations of 100K cells, 50K cells or 10K cells and the relative amounts of ATP were measured (Figure 3a). The ATP production levels at all cell concentrations were significantly lower in the J cybrid samples compared to the H cybrids: 100K group, 100±3.4% in H versus 62±3.9% in J, p<0.001; 50K group, 100±5.9% in H versus 59±4.7% in J, p<0.001; 10K group, 100±6.2% in H versus 40±4.9% in J, p<0.0001. In contrast, when the lactate levels were measured, the J haplogroup cybrids produced significantly higher levels compared to the H haplogroup (Figure 3b). When plated at a density of 100K cells, the J cultures showed a 1.8 fold increase in lactate production (3.57±0.15) compared to H cybrids (1.98±0.21, p = 0.0009). The J cultures plated with 50K cells showed 2.80±0.23 mM lactate concentration levels versus 1.78±0.13 in the H cybrid cultures, p = 0.008. Our findings indicate that the H haplogroup cybrids had lower levels of glycolysis than the J cybrids.

**Figure 4:**
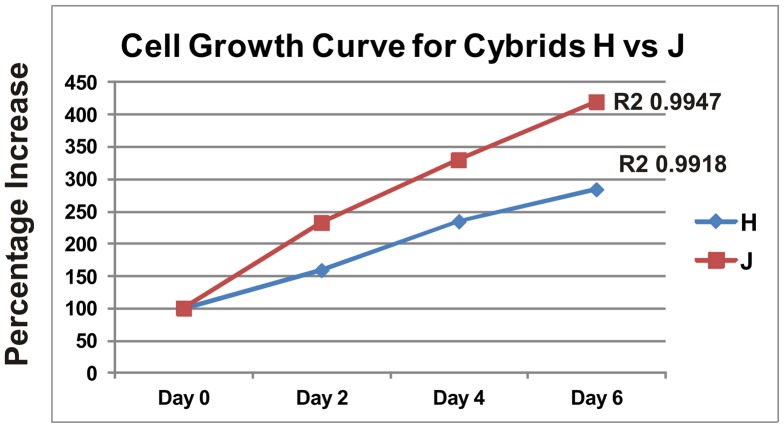
Six day growth curve shows a differential growth pattern for the H cybrids versus J cybrids. At Day 0, 30k cells per well were plated and the cell viabilities measured at Days 2, 4, and 6. The graph shows that the J cybrids grew at a faster rate than the H cybrids.

Cell growth was then compared for the cybrids. The H cybrids and J cybrids were plated with 30k cells per well, cultured over a 144 hour time period and then analyzed for cell viability at Days 0, 2, 4, and 6 (Figure 4). The Day 0 values were normalized to 100% in both H and J cultures. At each time point, the J cybrids showed greater growth than the H cybrids (Day 2, 232% vs 159%; Day 4, 329% vs 234%) and by Day 6 the J cultures showed 420% growth while the H cultures had 284% growth. Regression analyses showed the J cybrid cultures had greater growth rates than the H cybrid cultures. It was an unexpected finding that the H cybrids were producing more ATP but had lower growth rates compared to J cybrid cultures.

Not only is the influence of haplogroup important for cellular mechanisms, the total number of mitochondria can also significantly influence various pathways. The mtDNA copy numbers in the H cybrids and J cybrids were determined by Q-PCR using 18S to represent nuclear DNA (nDNA) and MT-ND2 to represent mtDNA (TaqMan, Life Technologies). An average of 3 independent representatives was used for each haplogroup. We found that at Day 1 the mtDNA copy numbers (nDNA∶mtDNA ratio) were similar in the H cybrid and J cybrid cultures (1.34±0.0228 versus 1.36±0.017, p = 0.59). At Day 7 the nDNA∶mtDNA ratios in the H and J cybrid cultures were 1.3±0.020 versus 1.26±0.016, p = 0.14, indicating that the mtDNA copy numbers were similar after the longer incubation period. Therefore, our results showed that the H cybrids and J cybrids had similar mtDNA copy numbers.

Based upon differences found between H cybrids and J cybrids in the growth studies as well as the energy pathway assays, we hypothesized that the H and J cybrids would have different expression patterns of the nuclear genes. Therefore, we isolated the RNA of the cultured three H cybrids (Cybd 10.07, Cybd 10.04, Cybd 10.03) and three J cybrids (Cybd 10.05, Cybd 10.01, Cybd 11.32), and pooled equal quantities from each sample The H cybrid RNA and J cybrid RNA were then analyzed using the Affymetrix array that characterizes the gene expression for over 40K genes. Analyses of the data using the INGENUITY systems statistical program showed that the predominant pathways altered in the H cybrids versus J cybrids were Cell Death, Cell-to-Cell Signaling, Growth and Proliferation, Cellular Movement, and Morphology (Table 1). The program also reported that the diseases and disorders affected by varying the mitochondrial haplogroups but having cells with identical nuclei were Cancer, Genetic Disorders, Dermatologic Diseases, Developmental Disorders, and Connective Tissue Diseases (Table 2).

**Table 1:** Molecular and Cellular Functions Associated with Gene Expression Differences Between H vs J Cybrids.

NAME	P-value	# Molecules
Cell Death	3.77E^−05^ - 2.59E^−02^	107
Cell-to-Cell Signaling & Interactions	6.86E^−05^ - 2.44E^−02^	55
Cellular Growth & Proliferation	2.61E^−05^ - 2.66E^−02^	159
Cellular Movement	6.56E^−08^ - 2.63E^−02^	117
Cell Morphology	2.64E^−05^ - 2.68E^−02^	52

**Table 2:** Diseases and Disorders Associated with Gene Expression Differences Between H vs J Cybrids.

NAME	P-value	# Molecules
Cancer	4.62E^−06^ - 2.63E^−02^	205
Genetic Disorders	3.60E^−05^ - 2.17E^−02^	182
Dermatological Diseases & Conditions	3.60E^−05^ - 2.03E^−02^	45
Developmental Disorders	3.60E^−05^ - 2.64E^−02^	56
Connective Tissue Disorders	3.60E^−05^ - 9.11E^−04^	13

**Figure 5:**
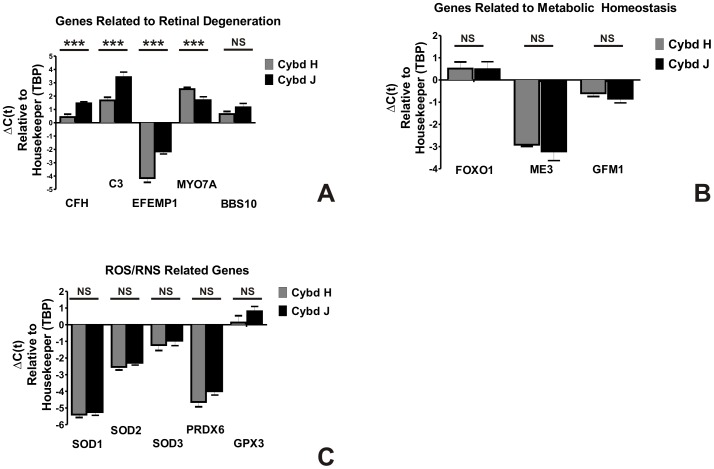
Q-PCR shows differential gene expression patterns for H cybrids versus the J cybrids. The relative delta C(t) values of the H cybrid cultures versus the J cybrid cultures are shown. Values are the average of three different H or J cybrids (originating from different individuals) run in six Q-PCR determinations. Error bars are SEM. *** p<0.001). The abbreviations for gene names are as follows: **Genes Related to Retinal Degeneration**. CFH, Complement Factor H; C3, Complement Component 3; EFEMP1, EGF-Containing Fibrilin-like Extracellular Matrix Protein 1; ARMS2, Age-Related Maculopathy Susceptibility 2; MYO7A, Myosin VIIA; BBS10, Chromosome 12, Open Reading Frame 58 for Bardet-Biedl syndrome. **Nuclear Encoded Genes**. FOXO1, Forkhead Box1; ME3, Malic Enzyme 3, NADP(+)-Dependent, Mitochondrial; GFM1, Mitochondrial Elongation Factor G1. **ROS/RNS Related Genes**. SOD1, Superoxide Dismutase 1; SOD2, Superoxide Dismutase 2; SOD3, Superoxide Dismutase 3; PRDX6, Peroxiredoxin 6; GPX3, Glutathione Peroxidase 3. NS, not significant; Cybd, cybrid; ROS/RNS, reactive oxygen/nitrogen species; TBP, TATA box binding protein.

We then performed additional in-depth analyses for genes of interest associated with AMD, ROS/RNS production and mitochondrial functions. For these studies, Q-PCR analyses were performed on individual H or J cybrid samples (n = 3 each) in triplicate. Figure 5 shows expression patterns for 14 different genes: (a) Six genes (CFH, C3, EFEMP1, MYO7A, BBS10, and ARMS2) that are reported to have high risk polymorphic associations with retinal diseases; (b) Five genes (SOD1, SOD2, SOD3, PRDX6, and GPX3) related to the production of ROS/RNS; and (c) Three nuclear encoded genes (FOXO1, GFM1, and ME3) that function in metabolic homeostasis. The Q-PCR showed that the J cybrids had decreased RNA expression levels for CFH (−1.0±0.8 ΔΔC_T_; 0.5 fold, p = 0.0001), C3 (−1.8±0.5 ΔΔC_T_; 0.29 fold, p = 0.0003), and EFEMP1 (−2.0±0.4 ΔΔC_T_; 0.25 fold, p<0.0001), compared to the H cybrids. The MYO7A gene expression was higher in the J cybrids compared to the H cybrids (0.83±0.29 ΔΔC_T_; 1.78 fold, p = 0.007). The BBS10 gene was expressed at similar levels in the H and J cybrids (p = 0.2). The ARMS2 expression levels were very low but similar in H and J cybrids (p = 0.89, data not shown). These Q-PCR findings were in agreement with the Affymetrix chip analyses which showed these genes to be elevated in the combined H haplogroup cybrid sample compared to the combined J cybrid sample. The antioxidant enzyme genes (SOD1 (p = 0.6), SOD2 (p = 0.3), SOD3 (p = 0.6), PRDX6 (p = 0.1), and GPX3 (p = 0.2) and nuclear encoded mitochondrial genes (FOXO1 (p = 0.9), ME3 (p = 0.4), and GFM1 (p = 0.3) showed no statistically significant differences by Q-PCR analyses between the H cybrid and J cybrid groups.

## Discussion

This study was designed to provide insights into the different molecular and functional outcomes of having the J haplogroup mtDNA variants versus the H mtDNA variants within cells that had identical nuclei. We found that the H and J cybrids have different modes of energy production. The H cybrids have higher levels of ATP production regardless of the plating density, indicating they utilize OXPHOS more effectively than the J cybrids. Elevated production of ATP levels has been reported in Huntington's patients that have the H haplogroup compared to non-H individuals [23]. Our analyses also showed that the H cybrids produced significantly higher levels of ROS/RNS compared to the J cybrids. Endogenous production of ROS occurs as electrons leak from the electron transport chain (ETC) within the mitochondria [24]. Our findings suggest that the J cybrids may have lower efficiency of the ETC leading to lower ATP and ROS levels which is similar to that described in osteosarcoma cybrids with J haplogroups [25]. Similar relationships between the H and J haplogroups have been reported in human subjects. Marcuello studied 114 healthy males and showed that those with the J haplogroup had lower maximum oxygen consumption (VO2max) rates than the non-J haplogroups [26], but that a steady state of exercise could eliminate this disparity [27]. Further analyses showed that this disparity was because the H haplogroup had significantly higher VO2max and oxidative damage than the J haplogroup individuals [28]. The inefficiency of the OXPHOS mitochondrial energy production found in J haplogroups may lead to lower ROS production and less oxidative damage, which in part may explain high correlations between centenarians and the J haplogroup population [29]–[31].

Using the cybrid model we found significantly higher lactate levels in the J cybrids indicating that these cells relied on glycolysis to a much greater degree than the OXPHOS-utilizing H cybrids. It has been shown in mitochondrial diseases (i.e., Leigh and MELAS syndromes) that diminished efficiency of the ETC is accompanied by higher lactate levels. Interestingly, when H cybrid and UK cybrid properties were compared, the UK cybrids had a 32% higher ATP production level and similar ROS levels compared to H cybrids [32]. This supports the idea that specific mtDNA SNPs that define the haplogroups can affect the cell energy function, pathway utilization and levels of oxidative stress.

In our study, comparison of the mtDNA to nDNA showed that the mtDNA copy numbers were similar in the H cybrids and J cybrids. We used 18S amplicon to represent the nDNA and MT-ND2 amplicon for the mtDNA. Suissa et al showed that cells with the J haplogroup backgrounds had increased mtDNA copy numbers compared to the H haplogroups [33]. The differences in the mtDNA copy numbers between Suissa and our studies may be related to different cell types for the Rho0 cells and the source of mitochondria. Their study used a Wal2 Rho0 cell line suspension which was then fused with chemically enucleated lymphoblasts containing either H or J haplogroups. In our study we used ARPE-19 cells which attach to the plate and the source of mitochondria was from platelets that lack nuclei and therefore, did not have to undergo chemical enucleation. In our study, with the mtDNA copy numbers similar in H cybrids and J cybrids, it implies that the changes found in ATP production and ROS formation were not simply due to lower numbers of mitochondria within the cells but were properties of mitochondrial haplogroups.

The differences in mtDNA SNP variants between H and J haplogroups are considerable. The H defining 7028C allele is a synonymous SNP located in the MT-CO1 gene. Further subtyping of the J haplogroups used in our study showed two cybrids were J1c and one was J1d1a. The J1c mtDNA has 14 SNP variants with 4 of those being non-synonymous, leading to amino acid changes in the MT-CYB, MT-ND3, and MT-ND5 genes compared to H mtDNA. The J1d1a variant has 15 SNPs with 3 non-synonymous changes in the MT-CYB, MT-ND3 and MT-ND5 genes. We believe these SNP variants could affect protein functions leading to altered ATP, lactate, and ROS production compared to the H cybrids. However, further work will be necessary to clarify the mechanisms.

The present study uses ARPE-19 cells, a cell line commonly used to analyze human RPE cell functions, as the background for cybrids to study mitochondrial function. Studies previously showed that removal of mtDNA in ARPE-19 cells caused significant changes in nuclear gene expression [21]. Moreover, osteosacrcoma cells with depleted mtDNA are resistant to chemically induced apoptosis [34]. This suggests that the absence or alterations of mitochondria can affect the expression of some genes. One goal of the present study was to investigate the influence of mtDNA variants on expression of genes important to development of AMD and to this end we utilized the Affymetrix chip assay with its large number of genes. Analyses of the H and J cybrids showed differences of 107 genes within the Cell Death pathways and 55 genes within the Cell-to-Cell Signaling pathways (Table 1). In terms of diseases that might be affected, the INGENUITY system analyses showed 205 genes from the Cancer category while 182 genes were associated with the Genetic Disorder category. At this time the mechanisms for these differences are not known. However, the H and J cybrids have identical nuclei and one can speculate that the influences may be within the nuclear-mitochondrial interactions, or possibly based upon energy requirements within the cells that could mediate pathway regulation. In either case, our findings show that mtDNA can affect changes in numerous important pathways and can mediate cell signaling and molecular functions. Our results indicate that 1) subtle changes in mtDNA can lead to a set of changes at the molecular and cellular level that are similar to those that occur with complete loss of mtDNA [21], [35], and 2) that these pathways changes are reminiscent of the retrograde response in yeast, C. elegans, Drosophila, mouse and human cells that is known to affect lifespan (in human cell population doubling levels; for review see [36], [37]).

Based upon the delta Ct values obtained from the Affymetrix array, we targeted three groups of genes for further analyses by Q-PCR:

### 1) Genes Related to Retinal Degeneration

Recent studies have demonstrated a close relationship between mitochondria, oxidative stress and inflammation. Complement activation and inflammation play a role in AMD, diabetic retinopathy and uveitis. The CFH and C3 genes are known high risk genes associated with AMD. In our study, the J cybrids had significantly lower expression levels of CFH and C3 than the H cybrids. The CFH gene polymorphism (rs1061170, T1277C, Tyr402His) has been associated with the AMD in Caucasian populations [38]–[42] but not Asians [43]–[45]. The CFH protein is a major regulator for the alternative complement pathway as it blocks C3 activation to C3b and causes C3b degradation. Using animal models, it has been shown that CFH is critical for long-term retinal function [46]. The CFH protein with the high risk Tyr402His polymorphism has a reduced capacity to bind with malondialdehyde (MDA), a lipid peroxidation cytotoxin, which could possibly contribute to a defective innate immune response for AMD patients [47]. In our study, the higher expression of CFH in the H cybrids would mean higher levels of the CFH inhibitor protein and may be a mechanistic way that the H haplogroup could be protective for AMD [19]. If the H cybrids have higher expression levels for CFH, then the products of the complement cascade should be lower, resulting in less oxidative stress, reduced inflammatory-related debris, and more protection for RPE cells.

The C3 gene expression was also lower in J cybrids (0.29 fold, p = 0.0003) compared to H cybrids. In Caucasian English and Scottish populations, sequencing studies showed that within exon 3 of C3, the common functional R102G polymorphism (rs2230199; 120700.0001) was strongly associated with AMD [48]–[50]. Studies show that AMD individuals have significantly elevated levels of C3a des Arg in their plasma compared with the age-matched control group, irrespective of the CFH polymorphism status [51]. In AMD eyes, drusen have an accumulation of complement-associated proteins including C3 [52], [53]. Cultured human RPE cells show a dramatically increased production of CFH and C3 after exposure to activated T cells [54] and oxidative stress induced by hydrogen peroxide [55]. It may be that the higher ROS production levels in the H cybrids stimulate upregulation of the complement components, including C3. Further studies will be needed to determine these interactions.

EFEMP1 is the third high risk AMD gene with lower expression in the J cybrids compared to H cybrids (0.25 fold, p = 0.0001). EFEMP1 is an extracellular matrix protein associated with drusen formation and complement activation in EFEMP1-R345W knock-in mice [56]. Histology of AMD eyes shows that EFEMP1 protein can be found between the RPE cell layer and drusen [57]. It has been suggested that misfolded EFEMP1 protein accumulates within RPE cells causing altered cellular function and inflammation. Higher EFEMP1 levels also play a role in tumor metastasis [58], [59]. ARMS2 was the fourth AMD-associated gene investigated. It has been suggested that expression of the LOC387715/ARMS2 gene yields a 12 kDa protein that localizes to the outer mitochondrial membrane [60], [61], although this has not been found by others [62]. In our study we found that the H cybrids and J cybrids have similar low levels of expression for ARMS2 (data not shown).

Our findings suggest that the mtDNA haplogroup defining variants mediate the expression of CFH, C3, and EFEMP1 genes and play a role in the inflammatory pathway of human RPE cells. The mechanism is not known at this time but one can speculate that the type of energy pathway, OXPHOS versus glycolysis, might feed back to the cell, influencing its innate immunity status. Studies using cybrids created from osteosarcoma cells have shown that mitochondrial haplogroups can influence the mRNA expression and intra-mitochondrial protein levels of HSP60 and HSP75, major elements in the stress responses for cells [63]. In addition, differences have been reported in expression for stress responsive nuclear genes interleukin-6, interleukin-1, and tumor necrosis factor receptor 2 [64]. Our findings suggest that cybrids with the H haplogroup mtDNA have different cellular homeostasis which may influence the response to oxidative stress, complement pathways, and degree of inflammation. This is significant because these responses are critical for the development of diseases, especially those related to the retina which has high metabolically active photoreceptor cells and is constantly exposed to UV light, both sources of oxidative stress. Another possible mechanism by which mtDNA variants may mediate cellular functions is through an as of yet unknown nuclear-mitochondrial DNA interaction(s) similar to that described for Saccharomyces cerevisiae cells grown under different metabolic conditions [65]. Studies show that mitochondrial dysfunction and damage can lead to a retrograde response that triggers many intracellular signaling pathways and nuclear genes [36]. The outcome is a compensatory adaptation that prolongs longevity (see excellent review [37]). No matter the mechanism occurring, our findings provide significant evidence that although the mtDNA is relatively small, in addition to energy production, it can mediate important cellular functions associated with inflammation which warrants further investigation.

Another gene investigated was MYO7A which was higher in J cybrids compared to H cybrids (1.78 fold, p = 0.006). This gene encodes an unconventional myosin that has structurally conserved heads but divergent tails to bind different macromolecules, allowing them to move along the actin filaments. Using this mechanism, the unconventional myosins can transport “cargo” within the cells [66]. The MYO7A gene is expressed in the RPE and photoreceptor cells, and has been associated with the retinal degeneration found in Usher syndrome type IB [67], [68]. In shaker-1 mice a mutation of the MYO7a gene caused abnormalities in the melanosome distribution in the RPE cells [69]. The RPE and photoreceptor cells are highly metabolically active and the different utilization of the energy pathways (OXPHOS vs glycolysis) of the H versus the J cybrids may affect the efficiency of the MYO7A protein and lead to cell death. Further work needs to be performed to understand the relationship between the bioenergetics of the cells and MYO7A protein function.

The BBS10 gene is related to the Bardet-Biedl syndrome, a rare autosomal recessive disease characterized by systemic abnormalities including progressive rod-cone retinal dystrophy, cognitive impairment, postnatal obesity, renal dysplasia, and polydactyly. In our study the expression of the BBS10 gene was similar in the H cybrid and J cybrid samples.

### 2) ROS/RNS Related Genes

Elevated ROS levels, such as seen in the H cybrid culture, can trigger activation or overexpression of various antioxidant genes. The SOD1, SOD2, SOD3 genes are important for conversion from superoxides into hydrogen peroxide, which is then converted by PRDX6 and GPX3 to water and oxygen. In our study, there were no differences in the expression levels for these antioxidant genes between the H and J cybrids. This is similar to results for H versus Uk cybrids which reported similar levels for MnSOD [32]. It may be that the levels of ROS produced by haplogroup-related SNPs are important for cell signaling but are not high enough to cause overexpression of antioxidant enzymes associated with pathological mutations or disease processes. It should be noted that in patients with osteoarthritis, the J haplogroup population did have higher levels of catalase, an antioxidant enzyme involved in the removal of hydrogen peroxide [70]. Furthermore, combining the haplogroup types with specific biomarkers allowed for phenotypic identification of osteoarthritis patients [71].

### 3) Genes Related to Metabolic Homeostasis

We found that three genes important for maintaining metabolic functions (FOXO1, ME3, and GFM1) had similar expression levels present in the H and J cybrids. This finding suggests that the haplogroup SNP variants do not mediate a generalized increase of expression of all metabolically-related genes, but rather elevation of specific pathways, in this case the inflammatory, stress responsive, and alternative complement pathways. This may be important for the retina, which is very metabolically active, and AMD, which has been associated with both mitochondrial dysfunction and inflammation.

**Figure 6:**
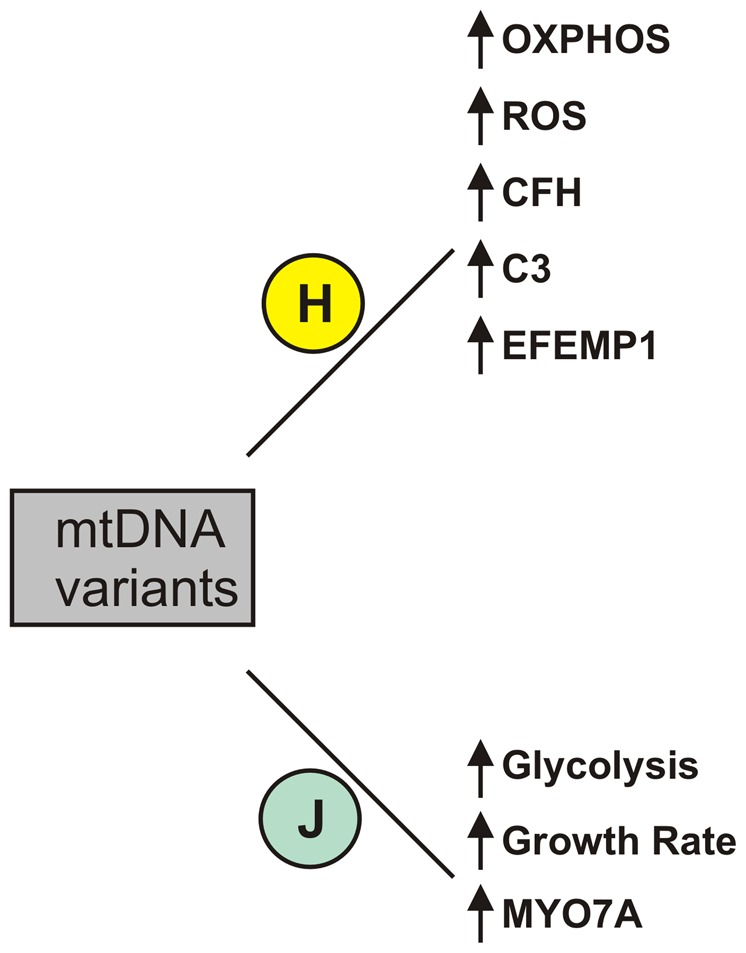
Schematic summary of the changes found in cybrids with H haplogroup mtDNA variants compared to cybrids with the H haplogroup mtDNA variants.

In summary, our findings demonstrate the valuable information that can be obtained using cybrid cell models. This study provides evidence that the mtDNA SNP variants associated with H versus J haplogroups can mediate differences in ROS, ATP and lactate production, influence cell growth rates, and modulate expression for genes involved in inflammation, oxidative stress, and apoptosis (Figure 6), which have been strongly associated with AMD. These findings are important because they provide possible mechanisms by which mitochondria-nuclear interactions can occur and they identify targets for possible drug interventions for AMD.
